# Correction: An investigation on the influence of catalyst composition, calcination and reduction temperatures on Ni/MgO catalyst for dry reforming of methane

**DOI:** 10.1039/d0ra90098b

**Published:** 2020-09-14

**Authors:** Muhammad Usman Rashid, W. M. A. Wan Daud

**Affiliations:** a Department of Chemical Engineering, University of Malaya 50603 Kuala Lumpur Malaysia usman_nfc@yahoo.com ashri@um.edu.my +60 379675319 +60 379675297

## Abstract

Correction for ‘An investigation on the influence of catalyst composition, calcination and reduction temperatures on Ni/MgO catalyst for dry reforming of methane’ by Muhammad Usman Rashid *et al.*, *RSC Adv.*, 2016, **6**, 91603–91616, DOI: 10.1039/C6RA15256B.

The authors regret that the name of one of the authors (Muhammad Usman Rashid) was shown incorrectly in the original article. The corrected author list is as shown above.

The Royal Society of Chemistry apologises for these errors and any consequent inconvenience to authors and readers.

## Supplementary Material

